# Tunable Tesla-Scale Magnetic Attosecond Pulses through Ring-Current Gating

**DOI:** 10.1021/acs.jpclett.3c02899

**Published:** 2023-12-06

**Authors:** Alba de las Heras, Franco P. Bonafé, Carlos Hernández-García, Angel Rubio, Ofer Neufeld

**Affiliations:** † Grupo de Investigación en Aplicaciones del Láser y Fotónica, Departamento de Física Aplicada, Universidad de Salamanca, Salamanca 37008, Spain; ‡ Max Planck Institute for the Structure and Dynamics of Matter and Center for Free-Electron Laser Science, Hamburg 22761, Germany; ¶ Center for Computational Quantum Physics, The Flatiron Institute, New York 10010, United States; § Nano-Bio Spectroscopy Group, Departamento de Física de Materiales, Universidad del País Vasco, San Sebastían 20018, Spain

## Abstract

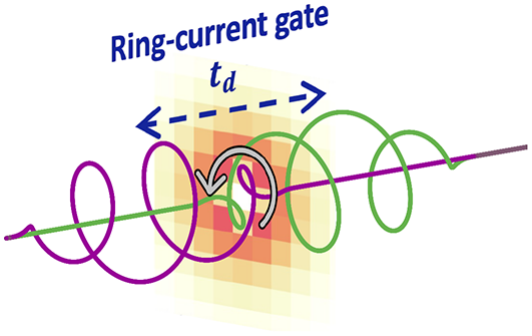

Coherent control over electron dynamics in atoms and molecules using high-intensity circularly polarized laser pulses gives rise to current loops, resulting in the emission of magnetic fields. We propose, and demonstrate with ab initio calculations, “current-gating” schemes to generate direct or alternating-current magnetic pulses in the infrared spectral region, with highly tunable waveform and frequency, and showing femtosecond-to-attosecond pulse duration. In optimal conditions, the magnetic pulse can be highly isolated from the driving laser and exhibits a high flux density (∼1 T at a few hundred nanometers from the source, with a pulse duration of 787 attoseconds) for application in forefront experiments of ultrafast spectroscopy. Our work paves the way toward the generation of attosecond magnetic fields to probe ultrafast magnetization, chiral responses, and spin dynamics.

Ultrafast photoionization occurs in the interaction of strong laser pulses with atoms or molecules. In the low-frequency regime, the dominant strong-field ionization mechanism is tunneling of bound electrons through the Coulomb potential barrier distorted by the laser field.^1,2^ The process of tunnel ionization has been extensively investigated in recent decades.^3−8^ Besides its fundamental importance for understanding ultrafast laser-induced electron dynamics,^9^ it plays a crucial role in high-order harmonic generation (HHG) and attosecond science in atoms and molecules.^10−12^ Tunnel ionization triggered by a circularly polarized pulse is an especially intriguing process, since the tunneling barrier rotates during the laser interaction, inducing additional angular momenta on both the bound and continuum electron wavepackets.^13−20^ As a result, the circularly polarized laser pulse induces a long-lived electronic ring current on the atomic scale,^17,21^ leading to the generation of a long-lived magnetic field of high intensity.^22,23^

The conventional generation of strong magnetic fields typically relies on conducting or superconducting materials arranged into loops or coils where the electric current flows.^24^ Such magnetic sources are particularly effective in quasi-static magnetic phenomena or long-duration magnetization dynamics.^25^ Also, more sophisticated strategies have been applied to improve the field strength, reaching up to 100 T in a magnetic texture.^26^ However, these approaches do not provide a straightforward way to generate ultrashort magnetic pulses, which could initiate and probe ultrafast spin responses, chirality, or magnetic dynamics at a high temporal resolution.

Alternatively, optical methods based on femtosecond laser pulses have made substantial progress in producing shorter magnetic bursts through laser-induced currents in plasmas,^27−30^ plasmonic nanostructures,^31,32^ semiconductors,^33−37^ molecules,^22,23,38−41^ or atoms.^42^ Interestingly, additional control over the magnetic field features can be gained by shaping the spatial structure of the laser beams.^29,30,34−37,43,44^ Nevertheless, to our knowledge the perspective of attosecond time scales has only been theoretically foreseen in relation to the attosecond electromagnetic pulses,^22,23,39^ where the dominance of the electric field overshadows the magnetic component. Therefore, the generation of isolated, strong, ultrashort magnetic fields is highly desirable to advance the control of purely magnetic phenomena, spintronics, chirotronics,^45−51^ or even HHG.^52,53^ Indeed, attosecond magnetic pulses would open up new avenues for the study and manipulation of magnetic phenomena on ultrafast time scales by accessing the fastest magnetic, spin, and chiral dynamics.^44,48,54−58^ For instance, magnetic sources reaching the attosecond scale could boost scientific and technological breakthroughs in ultrafast magnetometry, magnetic phase transitions, materials science, plasma physics, topological systems, and high-speed data storage. However, many challenges in obtaining bright ultrashort tunable magnetic pulses remain unresolved, such as addressing macroscopic effects, separating the magnetic pulse from the electric field, maintaining a high flux far from the target, and controlling the temporal durations and wavelength.

In this Letter, we propose laser-induced “current-gating” as a method to generate magnetic pulses within the femtosecond to attosecond regime. The long-lived electronic ring currents can be gated by using time-delayed counter-rotating circularly polarized laser pulses for high control over the pulse duration, waveform, and spectral content of the magnetic field emission. We demonstrate this proposal by performing ab initio calculations of time-dependent density-functional theory (TDDFT) coupled to Maxwell equations.^59,60^ This state-of-the-art theoretical approach allows us not only to analyze the ultrafast nonperturbative and out-of-equilibrium multielectron dynamics occurring during the laser–atom interaction but also the spatial and temporal profile of the magnetic field emission. We couple this ab initio single-emitter methodology with an analytic model describing the response of a full macroscopic gas jet, as employed in experiments. Our theory predicts that under a suitable choice of the driving parameters, the generated magnetic field can be isolated from the driving laser, showing a high flux density of ∼1 T at a few hundred nanometers from a macroscopic target. We further demonstrate that by employing sets of more than two gating pulses, the waveform of the generated magnetic pulse can be controlled to mimic a few-cycle pulse with the desired wavelength and duration. Our results set a route toward the tunable generation of Tesla-scale attosecond magnetic field pulses.

We first outline the system of study and our methodology to describe the laser–atom interplay. We explore the interaction of a train of intense circularly polarized laser pulses with an atomic noble gas jet in a collinear setup. The laser carrier wavelengths (800, 400, and 267 nm covering the infrared, visible, and ultraviolet regions of the electromagnetic spectrum) are chosen to be off-resonant with the typical energy scales of the atom. We are interested in high driving intensities that trigger highly nonlinear optical phenomena and strong-field ionization. We consider 4-cycle pulses full duration with a sinusoidal envelope, provided in the Supporting Information. Theoretically, the multielectron dynamics during the light–matter interaction are described ab initio within the time-dependent Kohn–Sham (KS) equations^61^ of TDDFT in the length gauge
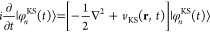
1in the open-access Octopus code.^60^ The equations are given in atomic units, discretized over a real-space grid, and solved in real-time. φ_*n*_^KS^(**r**,*t*) are the KS orbitals (with *n* being the orbital index), and **E**(*t*) is the external electric field in the dipole approximation (neglecting the magnetic field of the incoming laser pulse, which is a *c* factor weaker than the electric field). *c* is the speed of light in vacuum. The KS potential *v*_KS_(**r**, *t*) = *v*_ion_(**r**) + *v*_H_(**r**, *t*) + *v*_xc_[*n*(**r**, *t*)] – **r**·**E**(*t*) includes the usual terms describing the electron-nuclei interaction *v*_ion_ (which also incorporates interactions with core electrons), the Hartree potential *v*_H_, the exchange–correlation functional *v*_*xc*_ of the electron density *n*(**r**, *t*) = ,^61^ and the dipole term −**r**·**E**(*t*) describing the interaction with the laser field. We employ the adiabatic local density approximation including a self-interaction correction^62^ and the frozen-core approximation for inner electrons by using norm-conserving pseudopotentials^63^ for faster computational performance. The need for nonadiabatic exchange–correlation functionals in TDDFT to describe electron scattering processes^64,65^ or excitation mechanisms^66^ is of no relevance in the nonresonant generation of ring currents, where dynamical correlations are a minor effect and the physical process is well described by a single active electron picture.^13,14,17^ Within the TDDFT framework, we calculate the microscopic current density:
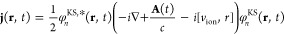
2In the dipole approximation, the vector potential, **A**(*t*), is a time-dependent function satisfying the following relationship with the external electric field: **E**(*t*) = . Considering the spatial degrees of freedom in the electromagnetic field is beyond the scope of this work. All additional details about the methodology and technical aspects can be found in the Supporting Information, where we also provide results from all-electron calculations or KS equations in the velocity gauge for further validation of our approach.

Among the noble gases, we focus the study on neon atoms due to their electron configuration 1s^2^2s^2^2p^6^, which constitutes the most compact distribution of closed-shell valence p-orbitals. Figure 1a illustrates the scheme of a single intense off-resonant circularly polarized electric laser pulse interacting with a neon atom. The figure shows the spatial profile of the current density obtained in our TDDFT calculations after the laser–atom interaction. The azimuthal current emerging in the vicinity of the nuclei is the consequence of asymmetric electron populations in *p*_+_ and *p*_–_ valence orbitals, which arise from a propensity in ionization rates from these orbitals under a circularly polarized driving laser.^13−17^ Curiously, the direction of the current flow opposes the helicity of the laser field because the ionization probability is higher for counter-rotating electrons than corotating electrons.^13−18^ Overall, the resulting magnetic field is linearly polarized along the light propagation axis, and its orientation depends on the clockwise or anticlockwise direction of the current. In Figure 1b, we present the dependence of the generated current amplitude on the driving peak intensity for different central wavelengths (267, 400, and 800 nm). Our results demonstrate that the azimuthal current increases for drivers with higher peak intensity and shorter wavelength. This follows the expected trend, since the mechanism behind the ring current generation relies on strong-field ionization, and its amplitude is proportional to the total ionized charge.

**Figure 1:**
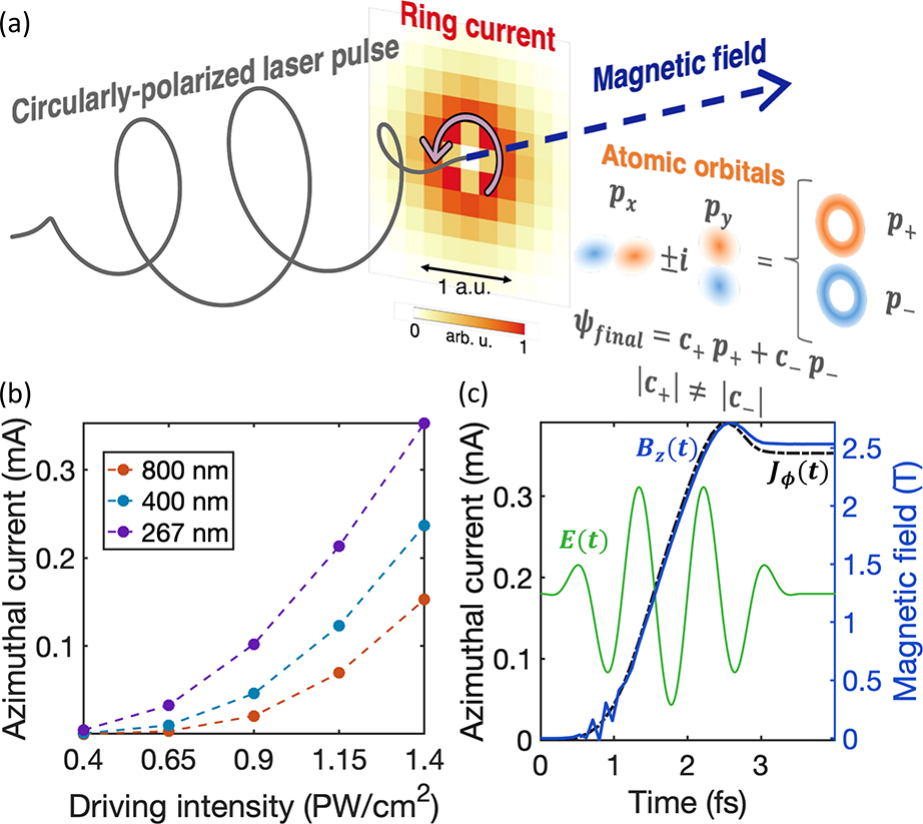
(a) Scheme of a circularly polarized electric pulse inducing a long-lived stationary azimuthal current, whose origin is associated with a different electron population in the *p*_+_ and *p*_–_ valence orbitals. The resulting magnetic field is static and linearly polarized along the longitudinal axis, and it persists after the laser pulse. (b) Dependence of the long-lived current amplitude on the driving peak intensity for different incident central wavelengths (267 nm is displayed in purple, 400 nm in blue, and 800 nm in orange). (c) The ultrafast buildup of the ring current (black dashed line) and the magnetic field (blue solid line) at the center of the current loop for a driving wavelength of 267 nm and peak intensity of 1.4 PW/cm^2^. We also depict in panel c the temporal profile of the incoming circularly polarized electric pulse (green line in an arbitrary vertical axis).

After reproducing the appearance of stationary ring currents with our theoretical approach, we explore their buildup and their connection to magnetic field emission. Differently from previous approaches to obtain the magnetic field based on particle-in-cell codes,^30,35^ the induced magnetic moment,^43^ or generalized Biot-Savart laws^36^ like the Jefimenko equation,^22,39^ we couple TDDFT and the microscopic Maxwell equations via the Riemann–Silberstein representation^59^ using the multisystem framework of Octopus.^60^ This formalism allows a reformulation of Maxwell’s equations in Schrödinger-like form, resulting in efficient coupled propagation in real-time and real-space of both the TDDFT KS equations and Maxwell’s equations.^59,60^

In our numerical multisystem coupling, the current density computed via TDDFT is the input for the Maxwell solver. Our approach neglects the nondipole magnetic field effects and the atomic back-reaction, whose effect is overshadowed by the strong driving electric field. We show in Figure 1c the ultrafast turn-on preceding the stationary regime for a driving wavelength of 267 nm and a peak intensity of 1.4 PW/cm^2^. Noticeably, we certify a direct correspondence between the azimuthal stationary current (black dashed line) and the magnetic field (blue solid line), as expected from the well-established Ampère–Maxwell law: ∇ × **B**(**r**, *t*) = . Practically, it allows us to simplify the following analyses by only computing the electronic current and generalizing the interpretations to the magnetic field. We refer to the Supporting Information for all numerical and technical details for this propagation scheme, as well as an in-depth analysis of the spatial distribution of the generated magnetic field. The temporal waveform of the driving circularly polarized electric pulse is depicted in arbitrary units (green line in Figure 1c) as a reference for readers.

Up until this stage, our results fully validate previous works on magnetic fields and impulses generated by strong-field-driven currents in other systems.^30,35,36,38−40,43^ However, as the currents are expected to be relatively long-lived, with electronic coherence times on scales of tens of femtoseconds that would only decay as the electron population in the valence orbitals equilibrates as a result of scattering processes, the magnetic field emission extends for several tens of femtoseconds, or longer. In order to generate shorter pulses, other strategies must be employed. In what follows, we demonstrate a current-gating strategy based on nonresonant optical switches for improved temporal control over the generated ring currents, thus creating an ultrafast magnetic pulse synthesizer to precisely shape the magnetic field emission on femtosecond to attosecond scales.

We propose the optical-gating configurations depicted in Figure 2a,b to control the temporal profile of the ring current (Figure 2c,d) and thus the waveform of the magnetic field. By combining two counter-rotating circularly polarized laser pulses separated by a tunable time delay (Figure 2a), we induce an ultrafast turn-on and subsequent extinction (Figure 2c) of both the ring-current and the magnetic field. To completely remove the ring current, the intensity of the second pulse needs to be adjusted to a slightly higher value than the first one (blue solid line in Figure 2c corresponds to *I*_1_ = 1.40 PW/cm^2^, *I*_2_ = 1.55 PW/cm^2^ at a central wavelength of 267 nm). This arises since the bound electron population depletes during the interaction, increasing the ionization potential of the system (binding the electrons more strongly), and thus the second pulse requires a higher intensity to balance the population of *p*_+_ and *p*_–_ orbitals.

**Figure 2:**
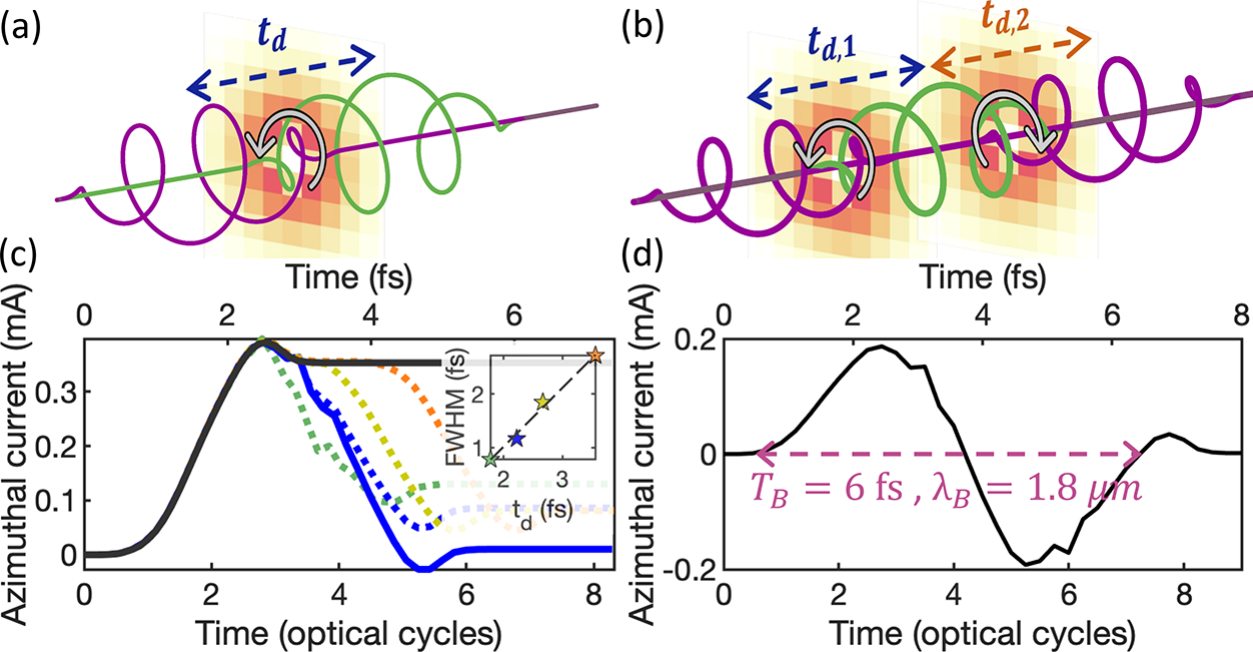
Current-gating schemes for waveform control of ultrashort magnetic pulses. (a) The synthesis of two time-delayed counter-rotating circularly polarized temporally gates the emission of a DC magnetic pulse by limiting the temporal window of the ring current. (c) The duration of the magnetic burst can be tuned by the time delay *t*_d_. The inset shows the linear relation of the full-width at half-maximum (fwhm) intensity of the magnetic field with the time delay, with the shortest pulse corresponding to 787 attoseconds. By adding a third gating pulse in panel (b), we synthesize in panel (d) the profile of a single-cycle magnetic pulse, whose wavelength can be tuned with the time delay between the pulses.

By tuning the time delay between the two pulses, *t*_d_, we shape the waveform of the magnetic field and gain control over the pulse duration, as emphasized in the inset of Figure 2c, reaching subfemtosecond duration (787 as). The full-width at half-maximum (fwhm) is defined in terms of the intensity of the magnetic pulse. Further progress in the development of high-intensity ultrashort driving pulses at shorter wavelengths could technically allow the generation of even shorter magnetic field pulses. Notably, without any additional manipulation, by setting longer time delays than the duration of the driving pulses, the magnetic field emission is temporally isolated, but they still copropagate and overlap in space. However, optical setups can be developed to filter out the driving laser field (both electric and magnetic components) to provide full spatial and temporal separation for applications. For example, by applying spectral filtering, our controlled magnetic field emission (which is DC in nature and carries much lower spectral content) can be isolated from the driver and other high-frequency emissions. We should also note that circularly polarized pulses in centrosymmetric isotropic media do not generate efficient high-harmonic emission,^67−70^ such that there is only a need to filter out the driving frequencies. Another option to spatially and temporally select the magnetic field emission is using polarization filtering, since the polarization of the synthesized magnetic field is orthogonal to the driver. Moreover, the interaction with a noble gas avoids the additional complexity of molecular alignment or ultrafast decoherence times due to ionic vibrations.^71^ All of these points should aid the experimental observation and application of our scheme.

Similar setups of collinear counter-rotating circularly polarized laser pulses are employed in polarization-gating techniques,^72,73^ which are widely applied to isolate a single attosecond pulse in HHG^69,74^ when the two pulses overlap in time. Even if the physical mechanism responsible for the attosecond electric field emission in polarization gating is different than here, these experimental developments^69,74^ also benefit the implementation for generating ultrashort magnetic pulses.

To provide further tunability in the magnetic emission, we consider a train of three gating pulses, as shown in Figure 2b. Remarkably, the central wavelength of the magnetic pulse can be controlled by tuning the time delay between the laser pulses. Figure 2d displays the temporal waveform of a single-cycle magnetic pulse whose wavelength of 1.8 μm (corresponding to a frequency of ∼167 THz) is determined by a time delay of 2.2 fs. The peak intensities of the 3 pulses are set to *I*_1_ = 1.0 PW/cm^2^, *I*_2_ = 1.4 PW/cm^2^, and *I*_3_ = 1.2 PW/cm^2^. By adjusting the peak intensities, pulse duration, and time delay of the drivers, we customize the temporal waveform of the magnetic field.

A closer inspection of Figure 2b,d points out that the ring current (and consequently the induced magnetic field) is maximum at the temporal gate between the two circular switch pulses (minimums of the driving electric field), which might appear counterintuitive at first glance. To clarify the physical mechanism behind this observation, we identify the role of each of the pulses in coherent control of the ring currents. The first pulse initiates the strong-field ionization asymmetry between *p*_+_ and *p*_–_ valence orbitals that gives rise to a ring current whose amplitude increases during the interaction. The second pulse, due to its counter-rotating circular polarization, first cancels this ring current and then builds up a current flow in the opposite direction (flipping the sign of the magnetic emission) since *I*_2_ is significantly higher than *I*_1_. Then, the third pulse (corotating with the first and counter-rotating with the second driver) is employed as a final optical switch to suppress the ring current. In essence, by tuning the time delay between a train of counter-rotating drivers, we effectively control the time window of the magnetic emission, which oscillates following the helicity of the laser. Note also that the wavelength of the magnetic field, λ_*B*_, is obviously determined by its period, *T*_*B*_, by the standard relation λ_*B*_ = *cT*_*B*_. In that respect, our scheme translates the time-delay parameter, which is easily tuned in experiments, to effective spectral-domain control over the magnetic pulse. It enables the temporal and spectral shaping of the magnetic field (including its carrier wave) by driving the system with off-resonant single-color laser fields. Hence, this approach should allow unique possibilities for exploring the responses of matter to variable frequency magnetic pulses. Importantly, by adding more switch pulses, one could in principle synthesize any desired magnetic field temporal waveform.

Finally, we extend the study to a macroscopic magnetic source composed of several ring currents by using an analytic model. This allows us to explore the expected magnetic field emission in the realistic conditions of the thin gas jets employed in experiments, whereas all results up to now considered only a single emitter. We sum the magnetic field of multiple stationary current loops placed on random positions separated by an average distance ⟨*d*⟩ (see Figure 3a,b), so that the longitudinal component is given by *B*_*z*_(**r**) = , where  is the loop index. For the averaged static magnetic component associated with each filamentary current loop of radius *a* = 0.2 au (which is taken from our ab initio TDDFT calculations), we consider the following off-axis equation:^75^
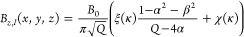
3where *B*_0_ is the magnetic field at the center of the loop, α =  and β = *z*_r_/*a* are the radial and longitudinal angles respectively defined in terms of the relative coordinates (*x*_r_, *y*_r_, *z*_r_) = (*x* – *x*_0_, *y* – *y*_0_, *z* – *z*_0_), whose origin lies at the center of the loop (*x*_0_, *y*_0_, *z*_0_). *Q* = (1 + α)^2^ + β^2^ and κ =  are dimensionless parameters. Then, χ(κ) and ξ(κ) are the complete elliptic integral functions of the first and second kind, respectively.^76^ Even though this model is limited by the assumption of steady-state independent ring currents and the coherence between all of the atoms in the gas, it should be a valid approximation at the peaks of the magnetic emission in our gating scheme (when the currents reach a stationary regime). Thus, our purpose is to obtain an approximation of the spatial scaling of the magnetic field under the macroscopic conditions of the experiments.

**Figure 3:**
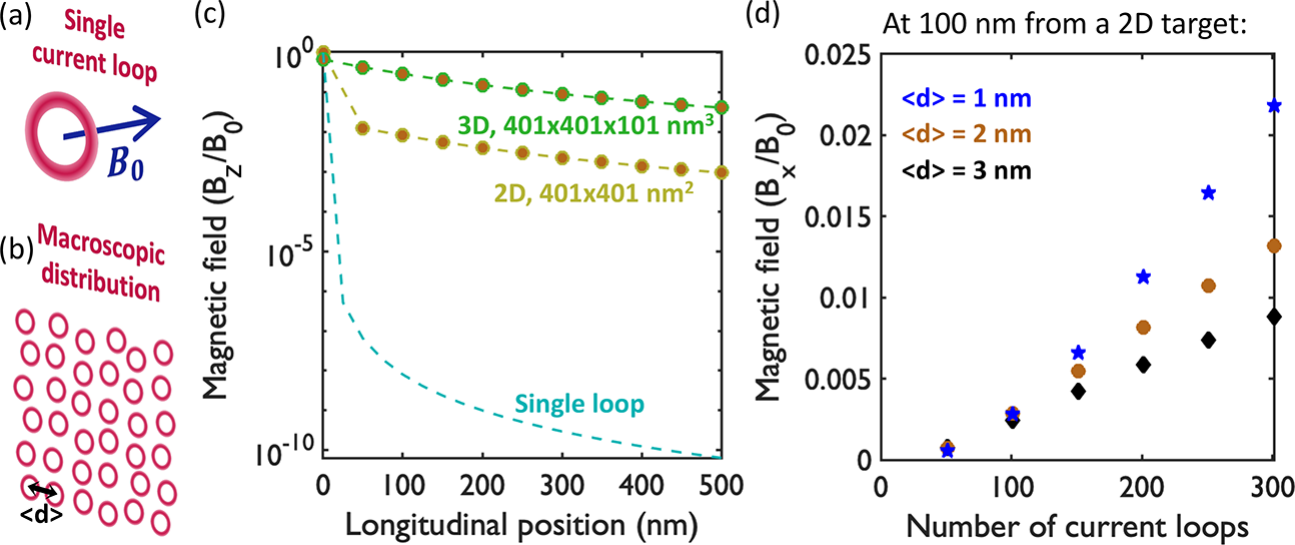
Illustration of (a) a single ring-current producing a magnetic field *B*_0_ at the center and (b) a macroscopic distribution. (c) Decay of the magnetic field (relative to the field at the origin) along the longitudinal axis for a single loop (cyan), a 2D loop distribution of 401 × 401 nm^2^ (yellow), and a 3D target of 401 × 401 × 101 nm^3^, which is experimentally accesible (green). (d) Magnetic flux at 100 nm from the edge of the 2D distribution as a function of the number of loops for different average separation distances ⟨*d*⟩.

In Figure 3, we analyze the decay of the macroscopic magnetic field in terms of *B*_0_. The magnetic field of a single ring current drastically decreases even for small distances along the longitudinal axis (cyan line in Figure 3c), which is trivially expected from the standard  dependence on the Biot-Savart law. In contrast, the decay is considerably diminished for a 401 × 401 nm^2^ array of current loops (yellow line in Figure 3c) or even more for a volumetric distribution of 401 × 401 × 101 nm^3^ (green line in Figure 3c, where the loops are separated by an average distance of ⟨*d*⟩ = 2 nm). This geometry mimics realistic accessible conditions for typical strong-field ionization experiments. From a magnetostatics perspective, one could imagine an approximately infinite wall of current loops, yielding a uniform field as long as the detector is placed at a longitudinal distance smaller than the size of the wall. This provides an intuitive explanation for why the magnetic field emission is still considerable even at a reasonable distance of a few hundred nanometers away from the gas jet.

This reduced spatial decay is particularly important for the applicability of such magnetic sources, since the sample can be positioned hundreds of nanometers from the edge of the magnetic source, instead of requiring atomic-scale precision. Figure 3d shows the magnetic flux at 100 nm from the edge of a 2D target as a function of the number of loops for different average separation distances (⟨*d*⟩ = 1 nm in blue, 2 nm in orange, and 3 nm in black). As expected, the magnetic flux increases with the ring-current density, i.e., larger number of loops and smaller separation distance. Importantly, for achievable experimental conditions, the expected magnetic flux at a distance of ∼100 nm from the gas jet can be as high as 1 T, while still generating pulses with durations of hundreds of attoseconds.

In summary, we propose to use a train of counter-rotating circularly polarized laser pulses to control the electron population imbalance in the *p*_+_ and *p*_–_ valence orbitals of neon atoms. These drivers can be viewed as optical switches to temporally shape the magnetic field emission. The extension of our proposed “current-gating” technique to other systems like molecules or solids should be straightforward, since the key idea is simply to confine the magnetic emission into an ultrashort temporal window. Thus, our scheme enables the synthesis of tunable strong magnetic fields of femtosecond to attosecond pulse duration, with the possibility of isolation from the electromagnetic driver in both space and time by applying spectral or polarization filtering. Such sources constitute an ultrafast magnetic switch to probe the fastest magnetic, spin, and chiral dynamics.^44,47,48,54−57^ Furthermore, extension to drivers in other spectral regimes (like mid-infrared or X-rays) could provide magnetic fields in the Terahertz range or at extremely high frequencies. We also show that despite the spatially localized magnetic field emitted from a single ring current, the total field from multiple emitters exhibits a diminished decay of hundreds of nanometers, which can be applied in ultrafast spectroscopy experiments and attosecond metrology.

As a potential outlook, we expect that our technique could also be employed for studying the electron dynamics induced and probed by the magnetic pulse in the form of magnetic spectroscopy. By measuring magnetic fields, one could infer attosecond electron dynamics, in analogy with standard X-ray attosecond experiments or HHG spectroscopies.^6,10,77^ We believe this would open interesting opportunities for exploring electron correlations and spin–orbit coupled dynamics in complex systems.^78−82^

## Supporting Information Available

Details about the methodology, results of the spatial distribution of the magnetic field from a single emitter, and a gauge comparison of the ring-current output with/without the pseudopotential approach (PDF)
