# Supplementary material for: Tunable Tesla-Scale Magnetic Attosecond Pulses through Ring-Current Gating
Source: J Phys Chem Lett. 2023 Dec 6;14(49):11160–7. doi: 10.1021/acs.jpclett.3c02899 (PMC10726360; doi:10.1021/acs.jpclett.3c02899)
Supplement: Supplementary file 1 — jz3c02899_si_001.pdf [file jz3c02899_si_001.pdf]

# Supporting Information:
# Tunable Tesla-Scale Magnetic Attosecond Pulses through Ring-Current Gating

Alba de las Heras,[1,*] Franco P. Bonafé,[2] Carlos Hernández-García,[1] Angel Rubio,[2,3,4,†] and Ofer Neufeld[2,‡]

[1]*Grupo de Investigación en Aplicaciones del Láser y Fotónica, Departamento de Física Aplicada, Universidad de Salamanca, Salamanca 37008, Spain*

[2]*Max Planck Institute for the Structure and Dynamics of Matter and Center for Free-Electron Laser Science, Hamburg 22761, Germany*

[3]*Center for Computational Quantum Physics, The Flatiron Institute, New York 10010, USA*

[4]*Nano-Bio Spectroscopy Group, Departamento de Física de Materiales, Universidad del País Vasco, San Sebastían 20018, Spain*

# I. METHODS

## A. Time propagation and magnetic field emission

We perform ab-initio calculations of the laser-atom interaction in the the multi-system framework of the OCTOPUS open-access code [1, 2]. The multielectron dynamics are modeled with time-dependent density-functional theory (TDDFT) by solving in real-time and real-space the time-dependent Kohn-Sham (KS) equations[3] in the length gauge of the dipole approximation:

$$i\frac{\partial}{\partial t}|\varphi_n^{KS}(t)\rangle = \left[-\frac{1}{2}\nabla^2 + v_{KS}(\mathbf{r},t)\right]|\varphi_n^{KS}(t)\rangle \tag{1}$$

The KS potential

$$v_{KS}(\mathbf{r},t) = v_{ion} + v_H(\mathbf{r},t) + v_{xc}[n(\mathbf{r},t)] - \mathbf{r}\cdot\mathbf{E}(t) \tag{2}$$

reproduces the multi-electron density $n(\mathbf{r},t) = \sum_n |\langle\mathbf{r}|\varphi_n^{KS}(t)\rangle|^2$ associated to the KS orbitals $\varphi_n^{KS}(\mathbf{r},t)$, being $n$ the orbital index. The KS potential includes the usual terms describing the electron-nuclei interaction $v_{ion}$ (which includes also an effective core-electron shell described with norm-conserving pseudopotentials[4]), the Hartree potential $v_H(\mathbf{r},t) = \int d^3r' \frac{n(r',t)}{|\mathbf{r}-\mathbf{r}'|}$, the exchange-correlation functional $v_{xc}$ in the widely-used adiabatic local density approximation including a self-interaction correction[5], and the dipole term of the electric field interaction $-\mathbf{r}\cdot\mathbf{E}(t)$. The equations are given in atomic units (a.u.).

The electric dipole approximation is a long-established approach for modeling strong laser-matter phenomena at non-relativistic electron energies and considering laser fields whose wavelength and spatial dependence are much larger than the atomic scale. In the dipole approximation, the magnetic field of the laser pulse is neglected.

For our calculations, the equation of the total electric field $\mathbf{E}(t)$ associated with the train of counterrotating circularly-polarized laser pulses is written as a sum over $m$, the number of pulses, with electric field amplitudes $E_m$, time delay $t_d$, wavelength $\lambda$, and a 4-cycle pulse


* albadelasheras@usal.es

† angel.rubio@mpsd.mpg.de

‡ ofer.neufeld@gmail.com

duration $\tau = 8\pi/\lambda$:

$$\mathbf{E}(t) = \sum_m \frac{1}{\sqrt{2}}(\mathbf{u_x} + i(-1)^m \mathbf{u_y}) E_m \sin\left(\frac{\pi(t-t_d)}{\tau}\right)^{\frac{3\left|\frac{\pi(t-t_d)}{\tau} - \frac{\pi}{2}\right|}{4}} \cdot \Theta\left(\frac{\tau}{2} - |t - \frac{\tau}{2} - t_d|\right) \cos\left(\frac{2\pi c}{\lambda} t\right). \quad (3)$$

In this expression, $\Theta$ denotes the Heaviside function, and the unitary vector $\frac{1}{\sqrt{2}}(\mathbf{u_x} \pm i\mathbf{u_y})$ determines the right or left circular polarization (where $i$ is the imaginary unit). The field amplitudes $E_m$ have been varied between the range of 0.10 a.u. to 0.20 a.u, corresponding to peak intensities within 0.40 PW/cm$^2$ and 1.40 PW/cm$^2$. The driving central wavelength has been set to 800 nm, 400 nm and 267 nm, corresponding to the emission of the standard Titanium-sapphire laser (800 nm), its second harmonic (400 nm), and third harmonic (267 nm). Thus covering the infrared, visible and ultraviolet regions of the electromagnetic spectrum.

For our study, the relevant observable is the microscopic current density:

$$\mathbf{j}(\mathbf{r}, t) = \frac{1}{2}\varphi_n^{KS,*}(\mathbf{r}, t)\left(-i\boldsymbol{\nabla} + \frac{\mathbf{A}(t)}{c} - i[V_{ion}, r]\right)\varphi_n^{KS}(\mathbf{r}, t), \quad (4)$$

where the dipole vector potential associated with the laser pulse is a time-dependent function upholding $\mathbf{A}(t) = -\int_0^\infty c\mathbf{E(t)}$. Concretely, in our setup of collinear circularly polarized laser pulses, the induced electronic current flows along the azimuthal direction, $\phi$, so that the important component is

$$j_\phi(\mathbf{r}, t) = j_x(\mathbf{r}, t)\cos(\phi) + j_y(\mathbf{r}, t)\sin(\phi). \quad (5)$$

This electronic current acts as a source term in the microscopic Maxwell equations to yield a magnetic field along the light propagation direction ($z$). Therefore, synchronously to the TDDFT approach, we numerically solve the microscopic Maxwell's equations:

$$\boldsymbol{\nabla} \cdot \mathbf{E} = \frac{\rho}{\epsilon_0}; \qquad \boldsymbol{\nabla} \cdot \mathbf{B} = 0 \quad (6)$$

$$\boldsymbol{\nabla} \times \mathbf{B} = \mu_0 \mathbf{j} + \frac{1}{c^2}\frac{\partial \mathbf{E}}{\partial t}; \qquad \boldsymbol{\nabla} \times \mathbf{E} = -\frac{\partial \mathbf{B}}{\partial t}, \quad (7)$$

where $\rho$, $\epsilon_0$, $\mu_0$, and $c$ are the charge density, the vacuum electric permittivity, the magnetic permeability, and the speed of light, respectively.

Our calculations are performed in the multi-system framework of Octopus[1] that uses the Riemann–Silberstein vector representation in *forward coupling*[2] (where only the incoming electromagnetic field is considered in the laser-matter interaction, thus neglecting the back-response that the electronic current could have on the electromagnetic field). This formalism is thoroughly described in section II of ref. [1], and chapters III and VI of ref. [2].

Both the KS and the Maxwell (MX) coupled solvers are numerically discretized over a cartesian grid with temporal steps of $\Delta t_{KS} = 0.01$ a.u. and $\Delta t_{MX} = 0.001$ a.u, spatial spacing $\Delta x_{KS} = \Delta y_{KS} = \Delta z_{KS} = \Delta x_{MX} = \Delta y_{MX} = \Delta z_{MX} = 0.2$ a.u., with spatial box length $L_{KS} = 50.0$ a.u., $L_{MX} = 53.2$ a.u. in each direction, using an imaginary absorbing potential of 20 a.u. width in the KS solver, and another absorbing boundary of 1.6 a.u. at the edges of the MX mesh. These parameters were chosen to guarantee the convergence and a consistent propagation.

### B. Macroscopic analytical model details

We extend our analysis to a macroscopic target composed of many ring currents in order to mimic the gas jets employed in experiments. Our analytic model for the longitudinal component of the total magnetic field, $B_z(\mathbf{r})$, considers multiple stationary independent filamentary ring currents placed on random positions and separated by an average distance $\langle d \rangle$, so that

$$B_z(\mathbf{r}) = \sum_{\ell} B_{z,\ell}(\mathbf{r}), \tag{8}$$

where $\ell$ is the loop index. The radius of the current loops is set to $a = 0.2$, which is roughly the value obtained in TDDFT. Then, the off-axis magnetic field of each ring current is given by [6]:

$$B_{z,\ell}(x, y, z) = \frac{B_0}{\pi\sqrt{Q}} \left( \xi(\kappa) \frac{1 - \alpha^2 - \beta^2}{Q - 4\alpha} + \chi(\kappa) \right), \tag{9}$$

where $B_0$ is the magnetic field at the center of the loop, $\alpha = \sqrt{x_r^2 + y_r^2}/a$ and $\beta = z_r/a$ are the radial and longitudinal angles respectively defined in terms of the relative coordinates $(x_r, y_r, z_r) = (x - x_0, y - y_0, z - z_0)$, whose origin is randomly fixed at the center of each current loop $(x_0, y_0, z_0)$. For a more compact equation, the dimensionless parameters $Q = (1 + \alpha)^2 + \beta^2$ and $\kappa = \sqrt{4\alpha/Q}$ are defined. The complete elliptic integral functions of the first and second kind[7], $\chi(\kappa)$ and $\xi(\kappa)$ respectively, are computed numerically. Overall, this

model enables a rough estimation of the spatial scaling of the magnetic field emission from a macroscopic target, which in our case simulates a gas jet, but it could be applied to other systems, such as nanostructures.

## II. ADDITIONAL RESULTS

### A. Results at different gauges and without pseudopotentials

We compare the results of the time-dependent KS equations in the length and velocity gauges of the dipole approximation. Formally, both approaches should be fully equivalent, but of slightly different numerical costs. In the velocity gauge, the analogous equations are the following:

$$i\frac{\partial}{\partial t}|\varphi_n^{KS}(t)\rangle = \left[\frac{1}{2}\left(-i\boldsymbol{\nabla} + \frac{\mathbf{A}(t)}{c}\right)^2 + v_{KS}(\mathbf{r},t)\right]|\varphi_n^{KS}(t)\rangle \tag{10}$$

$$v_{KS}(\mathbf{r},t) = v_{ion} + v_H(\mathbf{r},t) + v_{xc}[n(\mathbf{r},t)] \tag{11}$$

This comparison is performed for an 8 cycles pulse, 800 nm central wavelength, 1.40 PW/cm$^2$ envelope's peak intensity, $\Delta x_{KS} = \Delta y_{KS} = \Delta z_{KS} = 0.40$ a.u., and $\Delta t_{KS} = 0.40$ a.u. The results in Fig. 1(a) obtained in the different gauges show a good agreement before the maximum of the laser pulse, but there is a small $\sim 12\%$ discrepancy in the maximum value of the azimuthal current. This discrepancy arises due to the different analytical definitions of our input external fields, based on $E(t)$ or $A(t)$ (related by a time derivative or integral). Thus, the effect is associated with a difference in the carrier-envelope phase (CEP) of the pulse. Still, we can conclude that either the length or velocity gauges are a valid approach to describe the phenomenology.

We have also investigated the influence of using pseudopotentials to describe the inner electron core-shell (1s orbital for Ne), which greatly reduces the required computational resources. To test whether this could introduce any numerical artifact, we performed an all-electron calculation test for a 4 cycles pulse, 267 nm central wavelength, 1.40 PW/cm$^2$ peak intensity, $\Delta x_{KS} = \Delta y_{KS} = \Delta z_{KS} = 0.40$ a.u., and $\Delta t_{KS} = 0.40$ a.u. In Fig. 1(b) we observe that a similar evolution is captured, apart from numerical instabilities in the all-electron calculation, likely appearing due to the employed grid spacing, which is not

properly capturing the spatial structure of the 1S orbital. Such artifacts could be removed by employing tighter grid spacing. Nevertheless, we can infer from our test that the pseudopotential approach provides a proper description of the azimuthal current, and that core electron contributions are negligible.

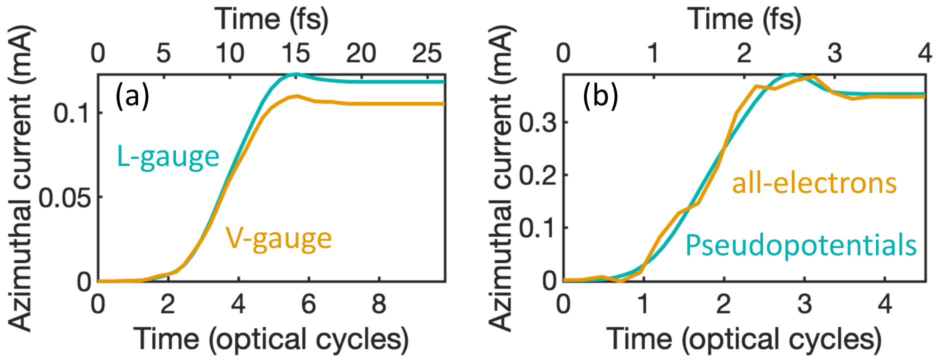


FIG. 1. Comparison of different theoretical models of the multielectron dynamics.

### B. Spatial distribution of the magnetic field from a single emitter

With our coupled KS-MX ab-initio approach, we study the interaction of a Neon atom with a driving laser pulse of 267 nm central wavelength at a peak intensity of 1.4 PW/cm$^2$, as in the main text. In the case of a single-atom emitter, the magnetic field is extremely localized. The spatial distribution of the longitudinal component, $B_z(\mathbf{r}, \mathbf{t})$, is shown in Fig. 2. The left and right panels show the dependence along the longitudinal and transversal axis respectively. The magnetic field is expressed in Teslas, whereas the spatial coordinates are given in a.u. We remark that the magnetic field shows an ultrafast turn-on and then it persists after the interaction with the laser pulse. The driving electric field is superimposed in the figures (grey line) for reference. As discussed in the main text, this magnetic field can be temporally confined and modulated by using a train of counterrotating circularly-polarized pulses. Still, the magnetic field's spatial degrees of freedom are preserved in all those cases.

Another interesting observation is that the driving laser also causes an emission of a weaker transversal circularly-polarized magnetic field component (see Fig. 3) that oscillates with the driving frequency of the laser. However, we keep in mind that these components

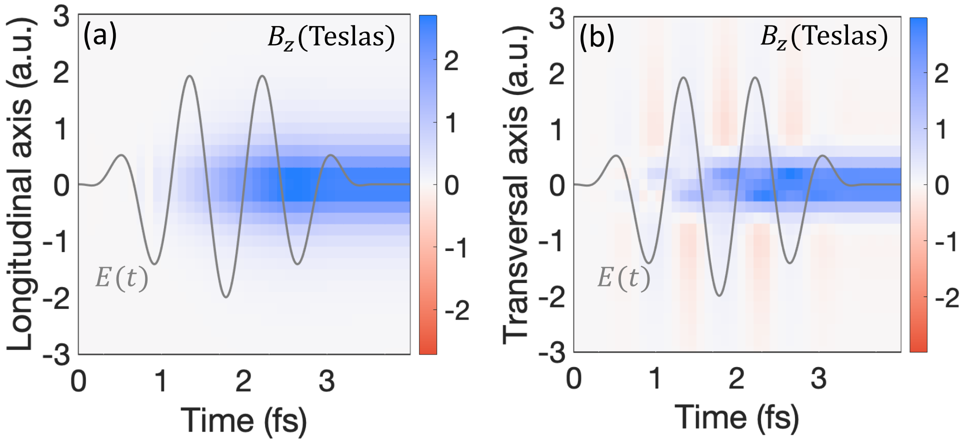


FIG. 2. Longitudinal component of the magnetic field $B_z(\mathbf{r}, t)$ along (a) the longitudinal axis and (b) the transversal axis during the interaction driving laser pulse (shown in grey line in arb.u.) at 1.4 PW/cm$^2$ peak intensity and 267 nm of central wavelength. Note that the magnetic field persists after the end of the driving pulse.

spatially and temporally overlap with the driving laser, and vanish when after the laser pulse.

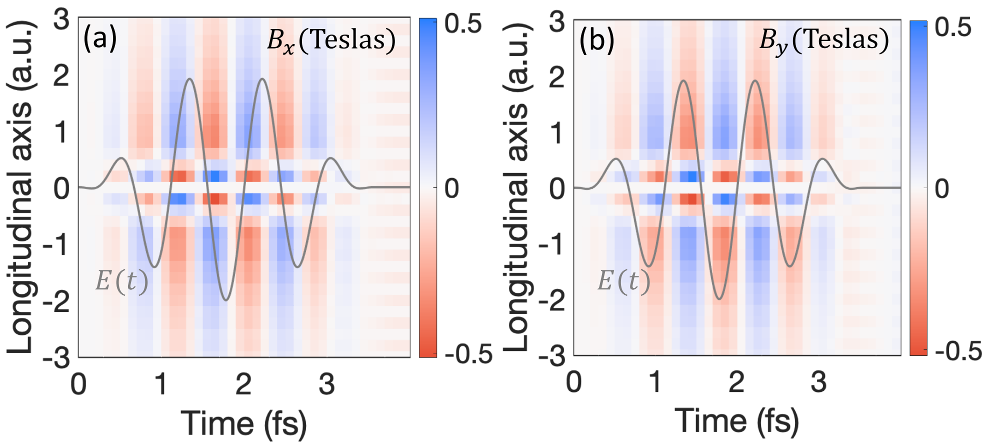


FIG. 3. Transversal magnetic field components dependence along the longitudinal axis: (a) $B_x(\mathbf{r}, t)$ and (b) $B_y(\mathbf{r}, t)$, during the interaction driving laser pulse (shown in grey line in arb.u.) at 1.4 PW/cm$^2$ peak intensity and 267 nm of central wavelength.

[1] Tancogne-Dejean, N.; Oliveira, M. J.; Andrade, X.; Appel, H.; Borca, C. H.; Le Breton, G.; Buchholz, F.; Castro, A.; Corni, S.; Correa, A. A.; De Giovannini, U.; Delgado, A.; Eich, F. G.; Flick, J.; Gil, G.; Gomez, A.; Helbig, N.; Hübener, H.; Jestädt, R.; Jornet-Somoza, J.; Larsen, A. H.; Lebedeva, I. V.; Lüders, M.; Marques, M. A.; Ohlmann, S. T.; Pipolo, S.; Rampp, M.; Rozzi, C. A.; Strubbe, D. A.; Sato, S. A.; Schäfer, C.; Theophilou, I.; Welden, A.; Rubio, A. *J. Chem. Phys.* **2020**, *152*, 124119.

[2] Jestädt, R.; Ruggenthaler, M.; Oliveira, M. J. T.; Rubio, A.; Appel, H. *Adv. Phys.* **2019**, *68*, 225–333.

[3] Marques, M.; Ulrich, C.; Nagueira, F.; Rubio, A.; Burke, K.; Gross, E. K. U. *Time-Dependent Density Functional Theory, Lecture Notes in Physics*; Springer: Berlin, 2006.

[4] Hartwigsen, C.; Goedecker, S.; Hutter, J. *Phys. Rev.B* **1998**, *58*, 3641–3662.

[5] Perdew, J. P.; Zunger, A. *Phys. Rev.B* **1981**, *23*, 5048–5079.

[6] Knoepfel, H. E. *Magnetic Fields: A Comprehensive Theoretical Treatise for Practical Use*; Wiley: New York, 2000.

[7] Abramowitz, M.; Stegun, I. *Handbook of Mathematical Functions: With Formulas, Graphs, and Mathematical Tables*; Applied mathematics series; Dover Publications: New York, 1965.
